# Temperature and Copper Concentration Effects on the Formation of Graphene-Encapsulated Copper Nanoparticles from Kraft Lignin

**DOI:** 10.3390/ma10060677

**Published:** 2017-06-21

**Authors:** Weiqi Leng, H. Michael Barnes, Zhiyong Cai, Jilei Zhang

**Affiliations:** 1 Department of Sustainable Bioproducts, Mississippi State University, Mississippi, MS 39762, USA; wleng@fs.fed.us (W.L.); mike.barnes@msstate.edu (H.M.B.); 2 U.S. Department of Agriculture, Forest Service, Forest Products Laboratory, Madison, WI 53726, USA; zcai@fs.fed.us

**Keywords:** temperature, copper concentration, graphene-encapsulated copper nanoparticles

## Abstract

The effects of temperature and copper catalyst concentration on the formation of graphene-encapsulated copper nanoparticles (GECNs) were investigated by means of X-ray diffraction, Fourier transform infrared spectroscopy-attenuated total reflectance, and transmission electron microscopy. Results showed that higher amounts of copper atoms facilitated the growth of more graphene islands and formed smaller size GECNs. A copper catalyst facilitated the decomposition of lignin at the lowest temperature studied (600 °C). Increasing the temperature up to 1000 °C retarded the degradation process, while assisting the reconfiguration of the defective sites of the graphene layers, thus producing higher-quality GECNs.

## 1. Introduction

Graphene-encapsulated copper nanoparticles (GECNs) have been extensively studied in the last decades because of their noble physical and chemical properties [1]. The core-shell structure GECNs enables its application in harsh environments because the graphene shell structure prevents oxidation of copper nanoparticles. Potential applications for GECNs are in wood and wood-based composite for their fungus and insect protection [2], electrical engineering [3], biomedical industry [4], etc. GECNs have been synthesized via many processes, including chemical vapor deposition (CVD) [5]. However, the formed graphene layers still have defects, and efforts have been made to improve the quality of the graphene structure. Epitaxial growth of graphene under a hydrogen gas environment showed an impressive improvement in graphene structure quality [6]. Hydrogen firstly helps to build an active surface of carbon, which is crucial for the subsequent graphene growth. Hydrogen also acts as an etching agent to eliminate defective sites and facilitates the growth of large-scale graphene [7]. Surface pretreatment can eliminate high protrusions and produce a smooth surface of the substrate and restore the defects of the substrate [8,9]. Surface pretreatment can also help dissolve the native oxide and passivate the substrate surface during graphitization [10].

Introducing a physical barrier is another effective way to improve the graphene layer structure quality. The idea situation is that carbon atoms start to grow into graphene right after the maximum process temperature is reached. Introducing an Al_2_O_3_ barrier between carbon source and metal catalyst retards the carbon diffusion process and reduces the pre-growth of graphene before reaching the maximum process temperature. The onset of growth is at a higher temperature when the thickness of the Al_2_O_3_ barrier is larger [11].

The configuration of heating chamber and the placement of metal catalysts also affect graphene growth. The one-end-close configuration restricts the carbon gas flow and consequently generates homogeneous graphene. The placement of metal catalyst affects the concentration of trapped carbon gas and subsequently determines the number of graphene layers formed [12]. The quality of the graphene structure can also be improved by controlling the concentration of carbon sources and reducing the cooling rate during CVD synthesis [13]. The concentration of carbon sources is proportionally related to the number of graphene layers formed. Reducing the cooling rate largely decreases the nucleation sites of graphene and makes it possible to grow large domains of graphene.

The synthesis of GECNs has been explained by different mechanisms. Recently, scientists have been inclined toward the dissolution–precipitation theory for metals with a large dissolution capacity for carbon, and toward the self-limiting theory for metals with a poor dissolution capacity for carbon, such as copper [14].

Polymers such as poly (methyl methacrylate) (PMMA), fluorine (C_13_H_10_), and sucrose (C_12_H_22_O_11_) can be solid carbon sources [15] for synthesizing GECNs. Limited literature was found related to the study of various factors on the formation of GECNs using lignin as a carbon source. Lignin is a byproduct from the pulp and paper industries and contains more than 60% carbon. Approximately 70 million tons of lignin are produced every year [16], mainly used as fuel. Lignin has many potential value-added applications, including the manufacture of carbon fibers and graphene [17].

It is known that the GECN properties such as surface morphology, structure, and crystallinity can be affected by many factors including the concentration of the copper catalyst [18] and the temperature [19]. However, the effect of the concentration of a copper catalyst on the GECN size distribution is unclear. Our previous study results [20] indicated that the formation of graphene layers surrounding copper nanoparticles can start at 400 °C. Copper nanoparticles were incompletely to near-completely shelled by graphene layers as temperature increased from 300 °C to 500 °C, and the graphene layers were formed by the self-limiting mechanism. This paper reports our continuing study on the effects of higher temperature levels (600–1000 °C) and the weight ratio of copper-to-lignin on GECN properties. In this study, the role that copper atoms play in the growth of graphene layers and the size distribution of GECNs was investigated. The relationships between temperature, the lignin degradation process, and the reconstruction of the graphene layers are also discussed here.

## 2. Materials and Methods

### 2.1. Materials

Deionized water purified BioChoice Lignin (BCL-DI) (Domtar Corp., Plymouth, NC, USA) was used as the carbon source. The metal catalyst, copper sulfate pentahydrate (CuSO_4_·5H_2_O), and nitric acid (HNO_3_) for purification were procured from Sigma-Aldrich.

### 2.2. Experimental Design

Experiment #1 was a 3 × 2 factorial experiment with three replicates per combination to evaluate temperature effects on the crystallinity of formed GECNs. The two factors were temperature (600, 800, and 1000 °C) and copper-to-lignin weight (oven-dried) ratio (0:1 and 1:4). The second experiment evaluated the copper lignin weight ratio effect (1:1, 1:2, and 1:4) at 1000 °C on the crystallinity of GECNs.

### 2.3. Precursor Mixing

All Cu-lignin samples were mixed using the following procedure: 3.9 g of CuSO_4_·5H_2_O (1 part of Cu by weight) and 12 g of BCL-DI (4 parts of lignin by weight) were first dispersed in distilled water, then heated at 80 °C, and stirred for 12 h followed by 24 h oven drying at 103 °C. Finally, the dried Cu-lignin mixture was ground well in an agate mortar before thermal treatment.

### 2.4. Thermal Treatment

Two porcelain boats, each holding 1.5 g of Cu-lignin mixture, were placed into the heating area of a 50 mm diameter, an 810 mm long quartz tube electric furnace (Lindberg/Blue M 1200) (Thermo Scientific™, Pittsburgh, PA, USA) equipped with a temperature controller (Lindberg/Blue UTC 150). Before heating, the air in the system was excluded by flowing argon gas for 15 min at a flow rate of 1800 standard cubic centimeter per minute (sccm). Then, temperature was raised to the target temperature level at a ramping rate of 20 °C/min and held at that temperature for 30 min. The heated Cu-lignin sample was cooled down naturally to ambient temperature under an argon atmosphere, and then transferred to a desiccator over CaCl_2_. The final weight of each sample was recorded, and its yield was calculated.

### 2.5. Characterization

All thermally treated Cu-lignin samples were purified with 20% HNO_3_ before characterization. For each trial, 0.5 g of GECNs was dispersed into 30 mL 20% HNO_3_ solution in a 125 mL of conical flask. The suspension was then heated up to the boiling point and kept boiling and stirring for 30 min. The suspension was filtered by a membrane (pore size: 0.45 µm) (VWR, Radnor, PA, USA) and rinsed with 550 mL of deionized water. The residue was dried in the oven at 60 °C for 6 h and then 103 °C overnight, weighed again, and stored in a glass vial for characterization.

X-ray diffraction (XRD) spectra was obtained from the X-ray diffractometer (Rigaku SmartLab, The Woodlands, TX, USA) utilizing Cu Kα radiation (*λ* = 1.5418 Å). The scanning range was from 10° to 90°, with a scan speed of 1°/min. Fourier Transform Infra-red Spectroscopy (FTIR) spectra of the powder samples were obtained using Thermo Scientific™ Nicolet™ iS™50 FT-IR Spectrometer (attenuated total reflection (ATR) probe). The spectra were recorded with 64 scans in the range of 4000–400 cm^−1^ and a resolution of 4 cm^−1^. High-resolution transmission electron microscopy (HRTEM) characterization was completed on a JOEL JEM-2100F. First, the sample was dispersed into acetone and sonicated for 15 min. One drop of the suspension was then dripped onto a 300 mesh copper grid with lacey carbon film (Agar Scientific) and air-dried overnight before characterization. The size distribution of nanoparticles was analyzed with the ImageJ software [21].

### 2.6. Statistical Analysis

A two-factor analysis of variance (ANOVA) general linear model (GLM) procedure was performed for Experiment #1 data to analyze the significances of two main effects and their interactions on carbonization yield. The protected least significant difference (LSD) multiple comparison procedure was performed to Experiment #2 data to analyze Cu-to-lignin weight ratio effects on carbonization yield and GECNs’ sizes. All statistical analyses were performed at the 5% significance level using SAS version 9.2 software (SAS, Cary, NC, USA).

## 3. Results and Discussion

The carbonization yield of each thermally treated sample was estimated by the following equation based on two assumptions: (1) there was no chemical reaction during the mixing of lignin and copper sulfate, and (2) copper element kept the same weight during the carbonization process, while sulfur and oxygen in gaseous forms were excluded from the system.

Y% = [(m_ma_ − m_b_) − (m_mb_ − m_b_) × 64/416]/[(m_mb_ − m_b_) × 256/416] × 100 (1)

where m_ma_ represents boat and sample mass after thermal treatment, m_b_ represents boat mass, m_mb_ represents boat and sample mass before thermal treatment, and 64/416 and 256/416 represent fractions of copper and carbon source in the mixture, respectively.

**Figure 1:**
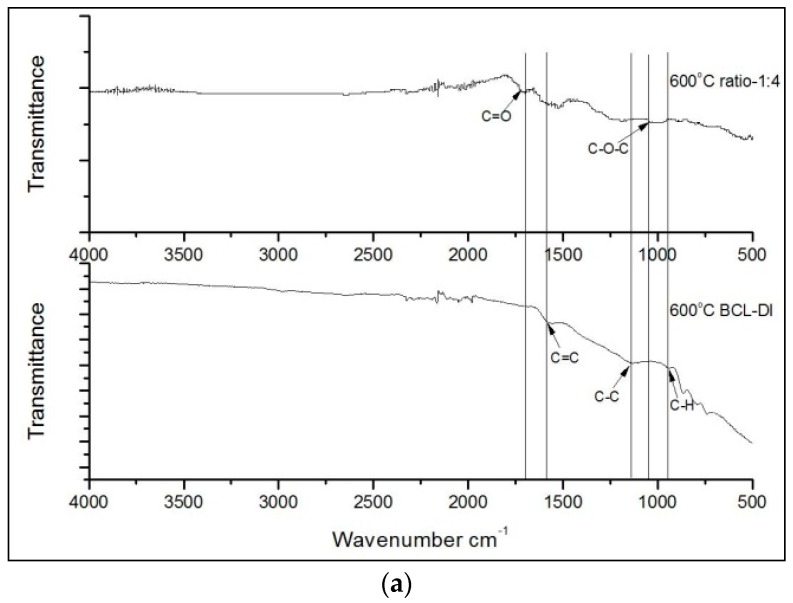

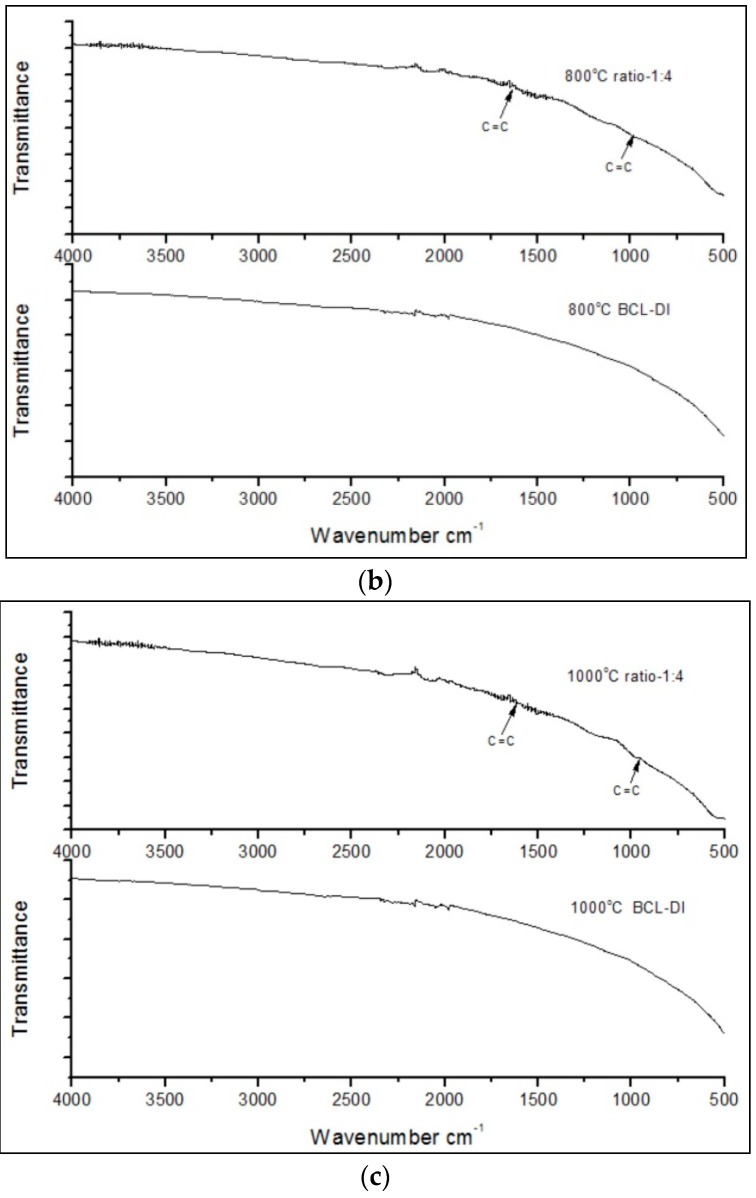
FTIR spectra of lignin samples with and without copper catalyst, treated at (**a**) 600 °C; (**b**) 800 °C; and (**c**) 1000 °C, respectively.

Table 1 summarizes mean carbonization yields of thermally treated samples with lignin alone and Cu-lignin mixture samples. The ANOVA results of Experiment #1 indicated that the two-factor interaction was not significant. Therefore, the main effect mean comparisons indicated that Cu-lignin mixtures had significantly higher carbonization yields than samples of lignin alone, which might imply that copper was not only decomposed the lignin, but also kept carbon in the mixture by forming graphene layers. This observation was supported by FTIR spectra (Figure 1), which indicated that when the Cu-lignin mixture was heated at 600 °C (Figure 1a), there were traceable oxygen-containing groups in the fingerprint region indicated by the aromatic C=O bond at 1700 cm^−1^ and the C–O–C bond at 1050 cm^−1^ [20]; however, weaker peak intensities of oxygen-containing groups were detected for pure BCL-DI lignin. These indicated that a faster degradation of BCL-DI lignin occurred and resulted in lower carbonization yields, while the addition of copper retarded the degradation process, i.e., a lower amount of lignin was decomposed into gases, thus resulting in higher carbonization yields. As temperature further increased to 800 °C and 1000 °C, there was no sign of function groups detected in the fingerprint area for pure BCL-DI lignin samples (Figure 1b,c). Peaks could be located around 1600 cm^−1^ and 1000 cm^−1^ for Cu-lignin samples, which indicated that graphene layers were detected. Both samples with lignin only and Cu-lignin mixture samples heated at 600 °C had significantly higher carbonization yields than 800 °C, followed by 1000 °C, which had the same trend as reported by Kim’s group [22]. 

LSD mean comparisons of yields in Experiment #2 indicated that the Cu-lignin mixture at a weight ratio of 1:4 had a significantly higher carbonization yield than the one of 1:2, followed by 1:1. This is the same trend as reported previously [23]. The reason was that more copper enabled a higher amount of lignin to be oxidized into carbonaceous gases vaporizing during the redox process of reducing copper ions to atoms and rendering the loss of carbon material.

**Figure 2:**
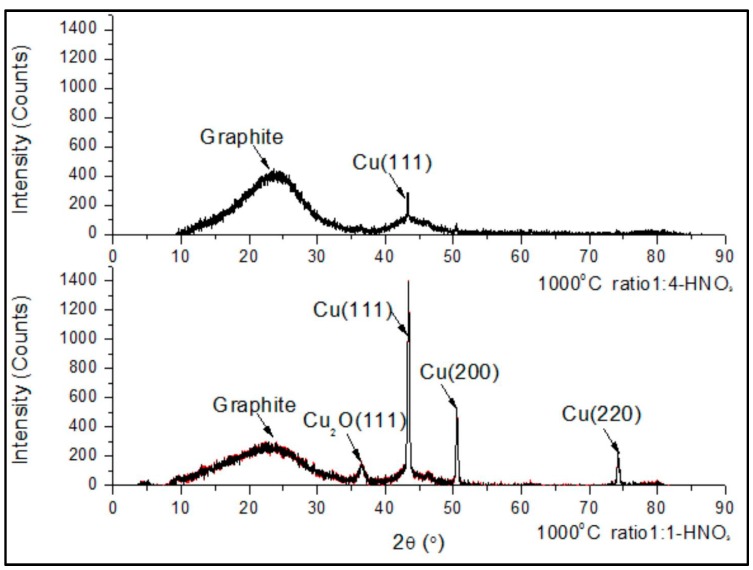
XRD spectra of Cu-lignin mixtures with their weight ratios of 1:4 and 1:1, respectively.

Figure 2 shows the XRD spectra of samples with two different Cu-to-lignin weight ratios treated at 1000 °C. The thermally treated sample with a higher Cu-to-lignin weight ratio of 1:1 had much higher intensity peaks at Cu (111), Cu (200), and Cu (220), respectively, than those with a lower Cu-to-lignin ratio of 1:4. In addition, the sample with a higher copper concentration had a sharp Cu_2_O peak, which was not found in that with a lower copper concentration. These indicated that a higher Cu-to-lignin weight ratio of 1:1 resulted in extra copper atoms uncovered by graphene layers and oxidized during HNO_3_ purification [12]. This implies that there is an optimum Cu-to-lignin weight ratio that will cause all copper nanoparticles to be shelled by graphene layers.

Typically, the sharper the graphite peak appears on a material XRD spectrum, the higher the crystallinity is [24]. However, the shape of the graphite peak is dependent on the copper element intensity. The graphite peaks around *2θ* = 24° (Figure 2) were still obvious compared to the high intensity of copper peaks, indicating that the graphite crystallinity of a higher Cu-to-lignin weight ratio was higher than the one with a lower weight ratio. This would be explained by the fact that the graphite peak can barely be detected in the XRD spectra because of the synergistic effect of the low crystallinity of graphite and the high intensity of the catalyst peak [25] if there is a limited amount of graphite in the sample. 

**Figure 3:**
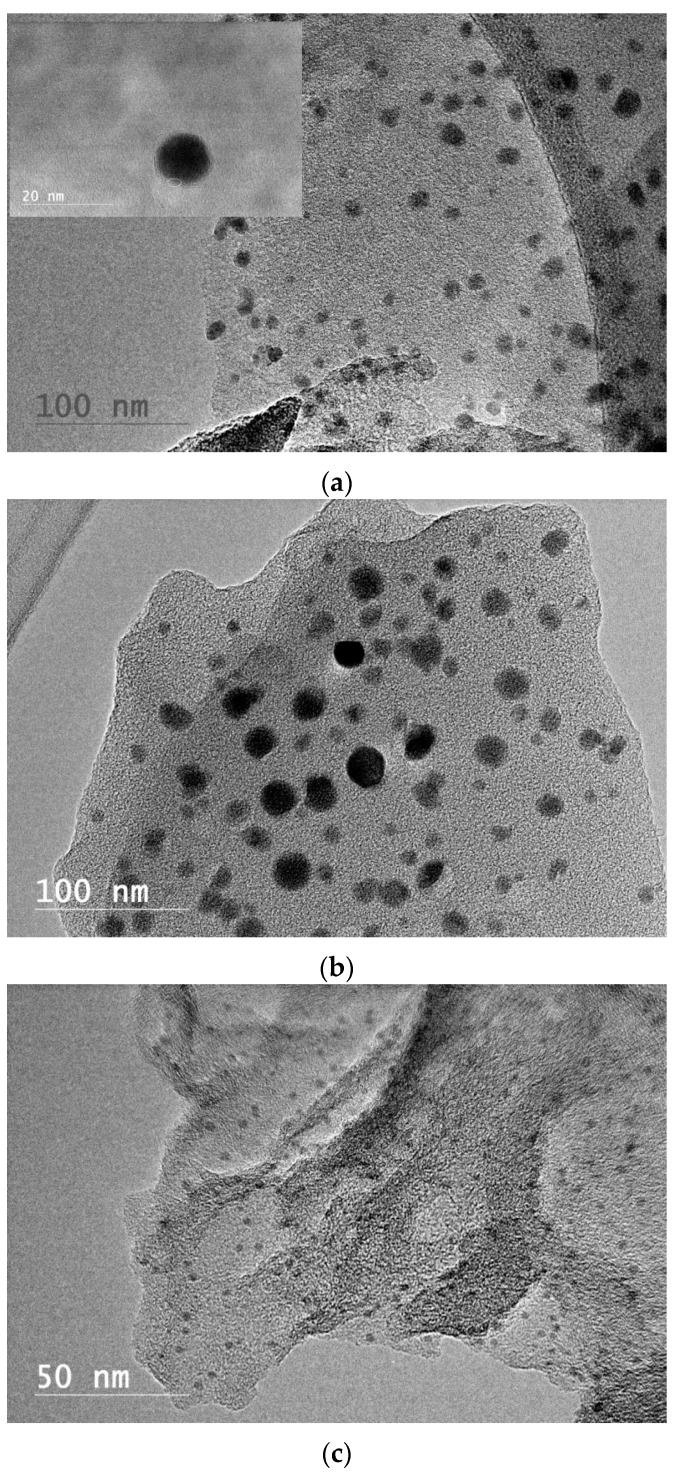
TEM images of Cu-lignin mixtures with weight ratios of 1:4 (**a**); 1:2 (**b**); and 1:1 (**c**), treated at 1000 °C.

HRTEM images (Figure 3) illustrate uniformly distributed GECNs with three different Cu-to-lignin weight ratios evaluated at 1000 °C. These GECNs’ diameters averaged 8.41 nm, 11.81 nm, and 1.54 nm with a coefficient of variation of 25.2%, 45.3%, and 22.5% for Cu-to-lignin weight ratios of 1:4, 1:2, and 1:1, respectively. Mean comparisons indicated that there were significant differences among three particle sizes. The particle size decreased significantly when the weight ratio increased from 1:2 to 1:1, while the particle diameter increased significantly when the weight ratio increased from 1:4 to 1:2. The particle size’s downward trend was similar to the observation from the study of mixing poly (vinyl alcohol) with iron citrate, with an iron-to-carbon weight ratio increasing up to 7:9 [23], and from another study wherein ferrocene and aromatic heavy oil was mixed with a ferrocene-to-oil weight ratio increasing up to 7:20 [26]. However, the extent of size decline was lower than the one with copper because iron has a carbon solubility much higher than copper, which can prevent carbon from effectively acting as a barrier to prevent the agglomeration of iron nanoparticles [27]. Hypothetically, larger amounts of copper atoms provide more nucleation sites, catalytically facilitating more graphene growth islands and the subsequent formation of smaller size particles. This hypothesis was different from the one in another study using graphene oxide to synthesize Fe_3_O_4_ nanoparticles [28], which proposed that the nucleation sites provided by the graphene oxide were constant. A possible reason was that the redox reaction occurred when lignin was used instead of graphene oxide, causing the nucleation mechanism to be different. Further study needs to be conducted to verify our hypothesis.

Our previous study [20] showed that the onset of growth of the graphene layer occurred at above 300 °C, and the GECNs formed at lower temperatures (<600 °C) had less than five graphene layers shelling copper nanoparticles, but there were still functional groups observed at 600 °C (Figure 1a), which indicated the existence of defects on graphene layers [29]. Experiment #1 indicated that increasing the temperature from 600 °C to 1000 °C did not increase the number of graphene layers due to the self-limiting synthesis mechanism. However, the crystallinity of graphene layers was improved with a superior layer structure (inset of Figure 3a). The disappearance of functional groups at 800 °C and 1000 °C (Figure 1b,c) also indicated that the structure of the graphene layers was superior because only the carbon network existed in the system, without interruptions by non-carbon atoms [29]. There must be a reconstruction of defective graphene layers and a reconfiguration of carbon atoms. Larger graphene domains were formed [29], and a higher GECN crystallinity was obtained. At a high temperature (1000 °C), the amorphous carbon can even be converted into graphene without the help of a copper catalyst [15,18,30].

## 4. Conclusions

The effect of the process temperature and the copper catalyst concentration on the synthesis of GECNs was investigated. The carbonization yield of the Cu-lignin mixture was higher than that of the pure lignin when the copper concentration was less than 50%. For samples treated at 1000 °C, the carbonization yield decreased with the increase in copper concentration. The crystallinity of graphite for the Cu-lignin mixture with higher concentrations of copper was higher than that with lower concentration of copper. Larger amounts of copper atoms facilitated the growth of more graphene islands and formed smaller size particles. The copper catalyst retarded the degradation of lignin at temperatures ranging from 600 °C to 1000 °C while assisting the reconfiguration of the defective sites of the graphene layers, producing high crystalline graphene structures shelling copper nanoparticles.

## Figures and Tables

**Table 1:** Yield results for different treatment combinations.

Weight Ratio	Temperature (°C)		
	600	800	1000
0:1	41.47% (1.2%) ^1^	38.24% (0.7%)	37.30% (1.4%)
1:4	49.42% (2.6%)	48.02% (0.3%)	46.57% (0.5%)
1:2	-	-	42.78% (1.3%)
1:1	-	-	33.76% (2.1%)

^1^ Coefficient of variation

## References

[1] Host J.J., Dravid V.P., Teng M.H. (1998). Systematic study of graphite encapsulated nickel nanocrystal synthesis with formation mechanism implications. J. Mater. Res..

[2] Kartal K.N., Green F., Clausen C.A. (2009). Do the unique properties of nanometals affect leachability or efficacy against fungi and termites?. Int. Biodeterior. Biodegrad..

[3] Zheng Q., Cai Z., Ma Z., Gong S. (2015). Cellulose nanofibril/reduced graphene oxide/carbon nanotube hybrid aerogels for highly flexible and all-solid-state supercapacitors. ACS Appl. Mater. Interfaces.

[4] Rodrigo D., Limaj O., Janner D., Etezade D., Abajo F.J.G.D., Pruneri V., Altug H. (2015). Mid-infrared plasmonic biosensing with graphene. Science.

[5] Li X., Cai W., An J., Kim S., Nah J., Yang D., Piner R., Velamakanni A., Jung I., Tutuc E. (2009). Large-area synthesis of high-quality and uniform graphene films on copper foils. Science.

[6] Cai T., Jia Z., Yan B., Yu D., Wu X. (2015). Hydrogen assisted growth of high quality epitaxial graphene on the C-face of 4H-SiC. Appl. Phys. Lett..

[7] Maeda F., Hibino H. (2013). Molecular beam epitaxial growth of graphene using cracked ethylene. J. Cryst. Growth.

[8] Sanbonsuge S., Abe S., Handa H., Takahashi R., Imaizumi K., Fukidome H., Suemitsu M. (2012). Improvement in Film Quality of Epitaxial Graphene on SiC(111)/Si(111) by SiH_4_ Pretreatment. Jpn. J. Appl. Phys..

[9] Hsieh Y.P., Wang Y.W., Ting C.C., Wang H.C., Chen K.Y., Yang C.C. (2013). Effect of Catalyst Morphology on the Quality of CVD Grown Graphene. J. Nanomater..

[10] Seo J.H., Kang B.J., Mun J.H., Lim S.K., Cho B.J. (2010). Effect of a surface pre-treatment on graphene growth using a SiC substrate. Microelectron. Eng..

[11] Weatherup R.S., Baehtz C., Dlubak B., Bayer B.C., Kidambi P.R., Blume R., Schloegl R., Hofmann S. (2013). Introducing Carbon Diffusion Barriers for Uniform, High-Quality Graphene Growth from Solid Sources. Nano Lett..

[12] Rümmeli M.H., Gorantla S., Bachmatiuk A., Phieler J., Geißler N., Ibrahim I., Pang J., Eckert J. (2013). On the Role of Vapor Trapping for Chemical Vapor Deposition (CVD) Grown Graphene over Copper. Chem. Mater..

[13] Reina A., Thiele S., Jia X., Bhaviripudi S., Dresselhaus M.S., Schaefer J.A., Kong J. (2010). Growth of large-area single- and Bi-layer graphene by controlled carbon precipitation on polycrystalline Ni surfaces. Nano Res..

[14] Zou Z., Dai B., Liu Z. (2013). CVD process engineering for designed growth of graphene. Sci. Sin. Chim..

[15] Sun Z., Yan Z., Yao J., Beitler E., Zhu Y., Tour J.M. (2010). Growth of graphene from solid carbon sources. Nature.

[16] Lora J.H. (2010). Utilization Opportunities for Biorefinery Lignins: An Industrial Perspective.

[17] Fang W., Yang S., Wang X., Yuan T., Sun R. (2017). Manufacture and application of lignin-based carbon fibers (LCFs) and lignin-based carbon nanofibers (LCNFs). Green Chem..

[18] Lee S., Hong J., Koo J.H., Lee H., Lee S., Choi T., Jung H., Koo B., Park J., Kim H. (2013). Synthesis of few-layered graphene nanoballs with copper cores using solid carbon source. Appl. Mater. Interfaces.

[19] Leng W., Barnes H.M., Zhang J., Cai Z. (2017). Effect of processing parameters on the synthesis of lignin-based graphene-encapsulated copper nanoparticles. Wood Fiber Sci..

[20] Leng W., Barnes H.M., Yan Q., Cai Z., Zhang J. (2016). Low temperature synthesis of graphene-encapsulated copper nanoparticles from kraft lignin. Mater. Lett..

[21] ImageJ. https://imagej.nih.gov/ij/index.html.

[22] Kim J.D., Roh J.S., Kim M.S. (2017). Effect of carbonization temperature on crystalline structure and properties of isotropic pitch-based carbon fiber. Carbon Lett..

[23] Bystrzejewski M., Klingeler R., Gemming T., Buchner B., Rummeli M.H. (2011). Synthesis of carbon-encapsulated iron nanoparticles by pyrolysis of iron citrate and poly (vinyl alcohol): A critical evaluation of yield and selectivity. Nanotechnology.

[24] Theivasanthi T., Alagar M. (2012). Konjac Bio-Molecules Assisted, Rod-Spherical shaped Lead Nano Powder Synthesized by Electrolytic Process and Its Characterization Studies. Nano Biomed. Eng..

[25] Mun S.P., Cai Z., Zhang J. (2013). Fe-catalyzed thermal conversion of sodium lignoslfonate to graphene. Mater. Lett..

[26] Li J., Song H., Chen X., Liang J., Zhang Y. (2009). Effects of preparation parameters on formation of carbon-encapsulated iron nanoparticles. Carbon Tech..

[27] Chiu C.C., Lo J.C., Teng M.H. (2012). A novel high efficiency method for the synthesis of graphite encapsulated metal (GEM) nanoparticles. Diamond Relat. Mater..

[28] Li X., Zhu H., Feng J., Zhang J., Deng X., Zhou B., Zhang H., Xue D., Li F., Mellors N.J. (2013). One-pot polylol synthesis of graphene decorated with size and density-tunable Fe_3_O_4_ nanoparticles for porcine pancreatic lipase immobilization. Carbon.

[29] Amaya R.O., Matsumoto Y., Guzman M.A.P., Lopez M.O. (2015). In situ synthesis of Cu_2_O and Cu nanoparticles during the thermal reduction of copper foil-supported graphene oxide. J. Nanopart. Res..

[30] Lin T., Wang Y., Bi H., Wan D., Huang F., Xie X., Jiang M. (2012). Hydrogen flame synthesis of few-layer graphene from a solid carbon source on hexagonal boron nitride. J. Mater. Chem..

